# Meta-Heuristics in Short Scale Construction: Ant Colony Optimization and Genetic Algorithm

**DOI:** 10.1371/journal.pone.0167110

**Published:** 2016-11-28

**Authors:** Ulrich Schroeders, Oliver Wilhelm, Gabriel Olaru

**Affiliations:** 1 Department of Educational Science, University of Bamberg, Bamberg, Germany; 2 Department of Psychology and Education, Ulm University, Ulm, Germany; Universita degli Studi di Catania, ITALY

## Abstract

The advent of large-scale assessment, but also the more frequent use of longitudinal and multivariate approaches to measurement in psychological, educational, and sociological research, caused an increased demand for psychometrically sound short scales. Shortening scales economizes on valuable administration time, but might result in inadequate measures because reducing an item set could: a) change the internal structure of the measure, b) result in poorer reliability and measurement precision, c) deliver measures that cannot effectively discriminate between persons on the intended ability spectrum, and d) reduce test-criterion relations. Different approaches to abbreviate measures fare differently with respect to the above-mentioned problems. Therefore, we compare the quality and efficiency of three item selection strategies to derive short scales from an existing long version: a *Stepwise COnfirmatory Factor Analytical* approach (SCOFA) that maximizes factor loadings and two metaheuristics, specifically an *Ant Colony Optimization* (ACO) with a tailored user-defined optimization function and a *Genetic Algorithm* (GA) with an unspecific cost-reduction function. SCOFA compiled short versions were highly reliable, but had poor validity. In contrast, both metaheuristics outperformed SCOFA and produced efficient and psychometrically sound short versions (unidimensional, reliable, sensitive, and valid). We discuss under which circumstances ACO and GA produce equivalent results and provide recommendations for conditions in which it is advisable to use a metaheuristic with an unspecific out-of-the-box optimization function.

## Introduction

The advent of large-scale assessment, but also more frequent use of longitudinal and multivariate approaches to measurement in psychological research, lead to an increased demand for psychometrically sound short scales. Kruyen, Emons and Sijtsma [1] screened six leading psychological journals for articles that dealt with short scales in a five-year time period and found 164 abbreviated tests (ca. 7% of all reviewed articles). Moreover, this trend is not limited to psychological research, but extends to neighboring disciplines such as sociology, education, and economics. The benefits are apparent in large-scale assessment, because even small reductions in test length results in great financial savings and presumably higher response rates of participants [2]. Other applications of short scales include longitudinal studies and experience sampling [3]. Thus, there is a high demand to construct short scales. Usually, short forms are derived from abbreviating existing scales. For example, the full *NEO Personality Inventory* (NEO PI) consists of 240 items [4], which was reduced to a 60-item version [5], a 44-item version [6] and super-short 10-item measures [7,8]. Such abbreviated versions have created substantial controversy about the tests’ reliability, validity, and classification consistency [1,9–11].

In the first part of this paper, we discuss criticisms against the compilation of short scales from a diagnostic point of view. More specifically, abbreviating a scale could: a) change the internal structure of the measure, b) result in poor reliability and measurement precision, c) deliver measures that cannot effectively discriminate between persons on the intended ability spectrum, and d) reduce test-criterion relations. Pragmatically, psychologists often apply naive strategies to shorten measures such as deleting items based on highest “alpha if item deleted” statistics or remove items with the lowest part-whole corrected item-scale correlation. In most cases these approaches are psychometrically inadequate because, among others, they do not consider the factor structure of the measure. In the second part of the paper, we introduce three selection strategies—a *Stepwise COnfirmatory Factor Analytical* (SCOFA) approach, an *Ant Colony Optimization* (ACO) algorithm, and a *Genetic Algorithm* (GA)—and apply them to construct short versions of a picture-based vocabulary test that was originally comprised of 89 items. The main research question is whether it is possible to construct short versions (with 25, 20, or 15 items) that are equivalent to the original on factor structure, reliability, discriminating power, and on the magnitude of correlations with other variables. On these points, we compare the quality and efficiency of the solutions derived by the different selection strategies.

### Potential Threats of Scale Shortening to the Psychometric Quality of a Measure

The decade-long dominance of *Classical Test Theory* (CTT) on the development of psychological measurement instruments is coming to an end [12,13]. Despite the similarities between CTT and contemporary psychometrics there are also crucial differences. Whereas in CTT the *test score* is focal, in the so-called *new psychometrics* [14,15] the *item score* is decisive [16]. As a consequence, latent variable modeling (and especially confirmatory factor analysis), which provides a comprehensive framework for testing measurement models, directs focus to the dimensionality of a measure. Reducing a measure’s item pool might affect the factor structure of the instrument. To counter this threat, most test authors righteously advocate to take into account both statistical information (e.g., item difficulty) and content information (e.g., expert rating on item content) in the derivation of short scales [11,17]. For unidimensional constructs with a narrow scope (e.g., figural reasoning) it might be appropriate to rely primarily on statistical information. Of course, considerations of test content and construct coverage still play a vital role in the process of item generation, but if a large item pool is automatically generated based on predefined rules [18] it seems sufficient to predominantly focus on statistical information. In contrast, for multidimensional measures it is also important that the relative weighting of the factors needs to correspond to the weighting used in the long version. Generally, the structure of a scale needs to correspond to the theoretically assumed structure of the construct and the fit of the measurement model has to meet psychometric standards—independent of the length of the measure.

Besides the dimensionality, a second key feature in test construction deals with concepts of reliability and measurement precision. Reliability is often framed as internal consistency and as such is confused with concepts of homogeneity and unidimensionality [19,20]. It is easily demonstrated that high values of internal consistency (i.e., Cronbach’s *α*) allow no conclusion about the underlying structure of the measure [19,21]. On the *group level*, reliability is defined as the ratio of true-score or trait variance to the variance of the observed score. Due to this sample-based characteristic, reliability can be understood as group-level measurement precision [22]. This concept of reliability is related to, but not equivalent to, measurement precision on the *individual level* [16]. Measurement precision on the individual level allows quantifying the (un)certainty with which inferences can be drawn based on a person’s test score. Shortening a psychological measure may affect both reliability at the group level and the individual level. It is a well-known fact that group-level reliability estimates in CTT are strongly influenced by test length [10]. This relationship is also expressed in the Spearman-Brown prophecy formula that can also provide an estimate of reliability if test length is reduced. Applying the Spearman-Brown formula presupposes essentially *τ*-parallel tests (i.e., equal factor loadings and residual variances), which is often not met in real data. The dependency between test length and reliability also exists for other reliability estimates such as Cronbach’s *α* and McDonald’s *ω*. To make matters worse, high values for reliability at the group-level are not synonymous with high measurement precision at the individual level. Even if short scales exhibit satisfactory reliability, simulation studies showed that decision quality in applied settings is alarmingly low when using short scales: For example, Emons, Sijtsma, and Meijer [9] demonstrated that the proportions of correct classifications based on short scales (with maximal 12 items) was at its best 50% even if the items had good discriminative power and locations (i.e., item difficulty) near the cut-scores. In practical terms, the high degree of measurement error that is associated with short scales threatens the accurate diagnosis in clinical settings as well as in personnel selection [10].

In connection with the precision of a measure, an additional aspect of test construction needs to be addressed. In many applied settings, it is desirable to discriminate within a specific range of the ability distribution, for example, at some predefined cut point in the distribution of reading ability to diagnose dyslexia. Therefore, it is important to examine whether a shortened measure loses its discriminative power in the intended ability range. If the test is not tailored to the needs of a specific group (e.g., disabled children), a measure should cover the whole distribution of the construct in question. It is important to keep in mind that short versions in general lose discriminative power at an individual level. Sijtsma [23] showed that removing items from a scale also affects the width of the confidence intervals (CIs). However, item elimination affects the test length more seriously than it affects the CIs, resulting in CIs of an abbreviated test form covering a larger proportion of the test length and thus leaving less potential to discriminate properly. As a consequence, when characterizing measurement precision for decisions on an individual level, CIs should be considered in relation to the overall test length [10].

A last point that is considered vital refers to the concept of validity, which has been criticized as fuzzy and a “catch-all category for a range of challenging psychometric problems” [24](p425). Validity is not easily to grasp, since it lived through several cycles of reformulation [25]. It started with the well-known definition that a test is valid to the degree that it “really measures what it purports to measure” [26](p14) over to the influential definition of Cronbach and Meehl [27](p282) that “construct validation takes place when an investigator believes that his instrument reflects a particular construct, to which are attached certain meanings” which is examined by embedding a measure into a nomological network. Messick (1989) focused on the interpretations derived from a test score and tied them to ethical and social consequences. Finally, Borsboom et al. [25] provided a simple reductive conceptualization: A test is valid if it measures an attribute that exists and if variations in the attribute causally produce variation in the measurement outcome. This realistic stance is accompanied by a shift from the traditional view of epistemology, meaning, and correlation to concepts of ontology, reference, and causality. Even though the discussion and evolution of the concept of validity is ongoing [12], in practical terms validity is still often assessed in the way Cronbach and Meehl proposed 60 years ago, that is, evaluating correlations with measures of equal and unequal measurement intention (i.e., convergent and discriminant validation). In this context, an abbreviated test form should maintain the same relations to other variables of similar or different scope than the long version.

### Metaheuristics in Scale Shortening

In constructing short scales, it is usually impossible to compute all possible models. For example, to construct a short scale with 25 of the original 89 items the complete computational solution would be comprised of 8,387,464,681,021,193,060,082 models (= (8925)). Often simple selection strategies are used to overcome this problem [1]: For example, a *Stepwise COnfirmatory Factor Analytical* (SCOFA) approach could iteratively remove the item with the lowest factor loading from the item pool. A comparatively new trend is the compilation of short scales by means of automatic optimization algorithms [28–31] that have been demonstrated to outperform simple selection strategies [32]. According to this perspective, short scale construction is a typical optimization problem, such as the well-known *knapsack problem* (“Choose a set of objects, each having a specific weight and monetary value, so that the value is maximized and the total weight does not exceed a predetermined limit”). In the given context, the respective question is how to select a fixed set of items from a long version that satisfies certain criteria (e.g., build a unidimensional scale with good model fit). Metaheuristics such as *Ant Colony Optimization* (ACO) and *Genetic Algorithms* (GA) can be used in order to solve such problems of combinatorial optimization [33,34].

Most metaheuristics are inspired by natural mechanisms such as evolution or the foraging behavior of ants or honeybees. Because these algorithms are rarely used in psychological research, we outline the procedures in a non-technical manner and provide additional information on the implementation in the method section. The ACO algorithm traces back to a series of observations and experiments with ants [35,36] in which they used a bridge construction, resembling a symmetrically distorted 8, to connect an ant’s nest with a food source. After an initial phase of oscillating behavior, the ants tended to choose the shortest path from the nest to the food source. The reason for this emergent phenomenon is that each ant leaves a chemical trace using pheromones. Because more ants pass on the shortest route per time unit, pheromones accumulate on this route, whereas they evaporate on less frequented routes. Higher levels of pheromone then attract more ants until the majority of ants follow the shortest route. In the context of short scale construction, different sets of items (= ants) are randomly drawn from the larger item pool of the long version. For each item set, a structural equation model is estimated and evaluated with respect to an optimization function (= shortest route) such as model fit and factor saturation. Similar to pheromones accumulating faster on quicker routes and attracting more ants, items of a specific item set that best meet the optimization criterion obtain higher probabilities to get selected in the next iteration. As the number of iterations increases, the pattern becomes more distinct, resulting in an efficient (but not necessarily the best) solution.

**Fig 1:**
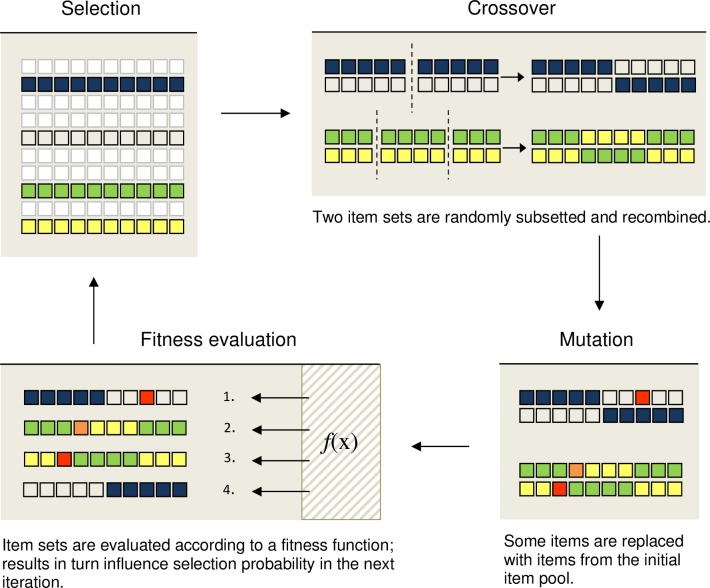
Illustration of the Genetic Algorithm. In the first iteration, the Genetic Algorithm randomly selects four item sets, which are highlighted in color, from an initial pool of 10 item sets. Each two of these sets are used to produce a new item set based on two principles: 1) subsetting and recombination (= crossover) and 2) random changes to the item set (= mutation). The newly assembled short versions are evaluated and ordered according to an optimization function (= fitness evaluation). The ordering influences the selection probability of an item to be assembled in a short version in the next iteration.

Genetic algorithms (GA) were first introduced by Holland [37] to solve computational problems of game theory and pattern recognition. GAs rely on the fundamental Darwinian evolution principles of selection, crossover, and mutation and try to mirror evolutionary processes. Most of the GA-terminology is borrowed from the field of genetics. In case of constructing a short scale with 10 items out of an item pool of 89 items, the procedure is as follows: In the first iteration, several item sets consisting of 10 items are randomly drawn from the item pool (see “selection” in Fig 1, see online supplement for a colored version). Items represent genes and item sets are analogous to chromosomes. Two of these item sets are selected to derive a new short form (equivalent to two parents produce an offspring) based on the following mechanisms: First, subsetting and recombining item sets (see “crossover” in Fig 1, in genetic terms: two chromosomes exchange one or more of their genetic sequences) and, second, random changes in an item set (see “mutation” in Fig 1, spontaneous changes in a genetic sequence). Mutated items are replaced with items of the initial item pool. The newly assembled short versions are then evaluated according to an optimization function (see “fitness evaluation” in Fig 1) and ranked based on their quality. If the result is better than the worst population member, the latter is replaced by the offspring (= survival of the fittest). This process is reiterated until a certain convergence criterion is met [38–40].

In comparison to stepwise selection mechanisms, item-sampling procedures feature several advantages: First, selection procedures that remove indicators stepwise are dependent on the sequence of items removed and are prone to local optima during the selection process. Metaheuristics on the other hand select fixed-sized item sets from the item pool, thus allowing for all possible combinations of items. Second, in contrast to traditional methods focusing on optimizing a single criterion, optimization through metaheuristics can be done with respect to several criteria simultaneously [29,32]. For example, it is possible to maximize the correlations with covariates while simultaneously retaining model fit.

## The Present Study

In the present study, we develop short scales based on an existing picture-based vocabulary test with 89 items that was administered to a large sample of secondary school students as part of the *National Educational Panel Study* in Germany [41]. We compare the quality and efficiency of the following three item selection strategies to derive short scales from an existing long version: *Stepwise COnfirmatory Factor Analytical* approach (SCOFA), *Ant Colony Optimization* (ACO), and a *Genetic Algorithm* (GA).

The SCOFA algorithm, which iteratively deletes the item with the lowest factor loading from the item pool, mirrors a current standard procedure of short scale construction [1]. In principle, stepwise procedures can be used to optimize different criteria such as item statistics (e.g., item difficulty) or fit indices [42]. The SCOFA approach implemented here is slightly more sophisticated than the often used simplistic strategy of discarding items in a single step based on the factor loadings of the full scale in an exploratory model, thus not accounting for model changes when items have been removed.

ACO algorithms mimic the foraging behavior of ants. Adoption of such algorithms can be suited to address the criticisms that common procedures of short scale construction neglect how reducing the item pool affects psychometric properties of the measure. The optimization function of the ACO algorithm as implemented here explicitly takes into account: a) overall model fit (to check for unidimensionality), b) reliability, c) discriminatory power at different points of the ability distribution, and d) changes in the relations with covariates, as convergent and discriminant validity. ACO draws item samples and iteratively increases the drawing probability of item combinations that meet the optimization function.

The second metaheuristic, GA, uses an unspecific cost reduction function that minimizes the number of items while explaining the maximum amount of variance of the original scale [40]. In contrast to ACO, which uses a theoretically-derived optimization function tackling important issues in short scale construction, GA utilize an unspecific out-of-the-box optimization function. Technically speaking, the complete search space for a long version with *L* items is 2^*L*^ and forms a so-called hypercube of *L* dimensions. The GA uses “hypercube sampling by sampling the corners of the *L*-dimensional hypercube” [43](p3). GA optimizes the search by mirroring evolutionary mechanisms (of selection, crossover, and mutation) while searching through the hypercube.

The main question for the three approaches is whether it is possible to compile short versions that maintain factor structure, are reliable and sensitive in the intended ability spectrum and show similar convergent and discriminant validity with other variables. Furthermore, we examine whether the metaheuristics (ACO and GA) outperform SCOFA and how the metaheuristics stack up against each other. Based on previous findings on the optimal test length [10], we restricted our analyses to short versions with 25, 20, and 15 items.

## Method

### Design and Participants

The data used in this study were collected in the *National Educational Panel Study (NEPS*). This study aims to describe educational processes and trajectories across the entire life span in Germany. NEPS is a large-scale multi-cohort sequence study that longitudinally follows six starting cohorts such as newborns, secondary school students, and adults [44]. We used data from 14,500 9^th^ Graders (starting cohort 4) that worked on a German adaptation of the picture-based vocabulary test. Mean age was 15.6 years (*SD* = 0.63; range 12.8–19.1 years); half of the sample was female (50.3%).

### Measurement Instruments

The *Peabody Picture Vocabulary Test* (PPVT) is an internationally widely used measure of receptive vocabulary and has been adapted into many languages [45]. In 2004, a German version of the PPVT with 204 items was constructed; based on an unspecified analysis, a subset of 89 items was selected and administered (referred to as PPVT-NEPS). In contrast to the original administration mode, the PPVT-NEPS has no criterion when to terminate testing. The task of the participants was to find the correct graphical representation of all words among four response alternatives.

In order to assess changes in the validity of the construct, we analyzed differences in correlations between scores of the long and the short scales with other variables. This set of variables is a small selection of the many variables gathered in NEPS and includes convergent (e.g., reading competence, reading speed) as well as discriminant measures (e.g., interest in and motivation to learn German). Thus, besides cognitive achievement (e.g., reasoning, math competence), motivational constructs (interest in and motivation to learn German) and sociodemographic indicators were included (e.g., age, sex). Table 1 gives additional information for all variables that were used to embed the PPVT-NEPS into a nomothetic network. For identification purposes, the original variable label is also listed.

**Table 1:** Description of Cognitive and Motivational Measures.

Covariate	Variable label	Description
Reading speed	rsg9_sc3/ rsci_sc3	Based on the test construction principles of the two *Salzburg Screening of Reading*, 51 sentences (with 5–18 words) were developed. After reading each sentence participants had to indicate whether the content of the sentence was “true” or “false”. Testing time was 2 minutes [46].
Math competence	mag9_sc1	Math competence was assessed in four content areas: a) quantity, b) space and shape, c) change and relationships, and d) data and chance. The test consisted of 22 items with a simple multiple-choice, a complex multiple-choice, or a short constructed response format. Testing time was 28 minutes [47].
Reading competence	reg9_sc1	The reading competence test assessed students’ abilities to find relevant information in a given text, draw text-related conclusions, and reflect on and evaluate these information. Students were asked to read 5 texts of different genres (informational, argumentative, literary, instructional, and advertising texts) and answer 5 item sets with a total number of 31 items. The response format of the items were mostly multiple choice. Testing time was 28 minutes [48].
Perceptual speed	dgg9_sc3a	Perceptual speed was assessed with a symbol-digit- test. Under severe time constraints participants had to assign the correct digit to the corresponding symbol. Secondary school students and adults worked on 93 items with a time limit of 90 seconds. [49].
Reasoning	dgg9_sc3b	Reasoning ability was measured with a matrices test; students were asked to detect the regularities by which geometric figures change and to choose a missing figure out of six possible response alternatives. The test included 12 matrices; testing time was 3 minutes [49].
Interest German	t66208a –t66208d	Students had to evaluate the extent to which statements regarding their interest in the German language applied to them (e.g., “I enjoy reading and writing texts. . .”) on a four-point rating scale (“does not apply at all”, “does not really apply”, “applies to some extent”, “applies completely”).
Motivation German	t66400a –t66400d	To assess student’s motivation in German, they had to evaluate four statements on a four-point scale ("does not apply at all", "does not really apply", "applies to some extent", "applies completely"). Example item: "I study in German class because I enjoy the subject matter"
German native language	t413000_g1D	Students select if German is their native language.
Grade German and math	t724101 and t724102	Refers to their grade on last year’s final report card in German/math classes, ranging in accordance with the German grading system from “very good (1)”, “good (2)”, “satisfactory (3)”, “passing (4)”, “poor (5)”, to “failing (6)”. In other words, lower values represent better grades.

Additional information on the items and constructs can be found in the codebook of the scientific user file (Leibniz Institute for Educational Trajectories, 2016) or through the references given at the end of the short descriptions. The second column gives the original variable labels in the data set.

### Statistical Analyses

Data preparation, recoding, and analyses were conducted with *R 3*.*2*.*0* [50]; CFA models were estimated with the R package *lavaan 0*.*5–20* [51]. The ACO script is a revised and an adopted version of the script provided by Leite (2015). The Genetic Algorithm is part of the R package *GAabbreviate 1*.*2* [43] which is an implementation of the routine suggested by Yarkoni [40]. Both the ACO and the GA script are available from the author’s website [52].

#### Stepwise confirmatory factor analysis (SCOFA)

The first method of item selection is a simple stepwise approach. After estimating a CFA for the original item set of 89 items, the item with the lowest factor loading is removed. The model is then re-estimated with the reduced item set and again the item with the lowest factor loading is removed. This procedure is repeated until the predetermined number of items for the short version is reached (i.e., 25, 20, 15). The CFAs were estimated with the *Weighted Least Squares Mean and Variance adjusted* (WLSMV) estimator, which is superior to maximum likelihood estimation for artificially dichotomized and categorical data in terms of model rejection rates and appropriateness of the factor loadings [53]. Values of the *Comparative Fit Index* (CFI) ≥ .95 and values of the *Root Mean Square Error of Approximation* (RMSEA) ≤ .08 were taken as indication of good model fit [54].

#### Ant colony optimization (ACO)

The second method for short scale construction uses an optimization function addressing four measurement aspects: a) overall model fit to test for unidimensionality, b) reliability in terms of factor saturation, c) discriminatory power across the ability distribution, and d) changes in the relations to covariates.

With respect to model fit, we used a combination of the incremental fit index *Comparative Fit Index* (CFI) and the absolute fit index *Root Mean Square Error of Approximation* (RMSEA)—as proposed in the two-index strategy presentation [54]. Model parameters were logit-transformed in order to scale the value range between 0 and 1 and to differentiate most around a given cutoff value [29]. For example, values for the CFI above .95 correspond to a transformed pheromone level greater than .50. Small differences near the cutoff are weighted more heavily than values at the extremes.

$$\varphi_{CFI}=\frac{1}{1+e^{95-100CFI}}$$ (1)

The RMSEA cutoff value indicating good model fit was set to .05.

$$\varphi_{RMSEA}=1-\frac{1}{1+e^{5-100RMSEA}}$$ (2)

Both model fit indicators were averaged for the pheromone level concerning model fit:

$$\varphi_{Fit}=\frac{\varphi_{CFI}+\varphi_{RMSEA}}{2}$$ (3)

The second criterion in the optimization function dealt with the factor loadings as an estimate of reliability. In the psychological assessment literature, reliability at the group level was frequently equated with Cronbach’s *α*, which in turn has steered cautionary notes [19,20,55]. Cronbach’s *α* is only a specific reliability estimate that is bound to an essentially *τ*-equivalent measurement model (i.e., fixed factor loadings) [56]. However, these requirements are quite strict and are often not met in real data. McDonald’s *ω* [57] as an index of measurement precision [16] that represents factor saturation in a unidimensional factor model and relates the squared sum of the factor loadings to the sum of the residuals (see formula 4). In contrast to Cronbach’s *α*, McDonald’s *ω* is also suitable for *τ*-congeneric models (i.e., varying rather than fixed factor loadings). In the present case, we consider values greater than .90 as good values of reliability:

$$\varphi_{Rel}=\frac{1}{1+e^{9-10\omega }}\;with\;\omega =\frac{{\left(\sum_{\text{i}=1}^{\text{n}}\lambda_{\text{i}}\right)}^{2}}{{\left(\sum_{\text{i}=1}^{\text{n}}\lambda_{\text{i}}\right)}^{2}+\sum_{\text{i}=1}^{\text{n}}1-\lambda_{\text{i}}{}^{2}}$$ (4)

The third part of the optimization function refers to the sensitivity of the measure. Sometimes, researchers intend to discriminate with higher precision in a specific part of the ability distribution (e.g., in the upper part if one wants to detect highly gifted children). Accordingly, a short scale should match the measurement intention of the long scale. In the present case, the PPVT-NEPS is an unspecific screening instrument used to assess receptive vocabulary. Therefore, item difficulties of the abridged form should cover a broad range with a mean close to .625 (taking into account the guessing probability of .25) in order to obtain high discrimination across the ability distribution. An ideal distribution of item difficulties would match the (normal) ability distribution. We consider a mean item difficulty of .625 a good proxy to optimize the sensitivity of the measure. In contrast to the other pheromones, *φ*_*Sens*_ uses a quadratic term:

$$\varphi_{Sens}=-5{\left(M_{item}-.625\right)}^{2}+1$$ (5)

The last part of the optimization function probes the correlations of the short/long version with relevant covariates (e.g., sex, age, reading speed, math competence). If correlations of the short version do not deviate from the corresponding correlations of the long form, the correlation matrices would be identical. Taking into account the standard errors, a maximum of differences in the correlations │.03│ or below was considered a good representation of the long form:

$$\varphi_{Cor}=1-\frac{1}{1+e^{3-100\;max}}$$ (6)

The overall optimization function took into account all four pheromone trails with equal weight. That is, the function maximizes the sum of the four optimization criteria:

$$\max f(x)=\varphi_{Fit}+\varphi_{Rel}+\varphi_{Sens}+\varphi_{Cor}$$ (7)

Because ACO finds a suitable, but not necessarily the best solution, analyses were replicated three times with different seeds; results presented are the best (with the highest pheromone level) out of three.

#### Genetic algorithm (GA)

Like ACO, GA can also make use of a user-defined optimization or fitness function [39]. In the present case, however, we applied the default implementation provided in the R package *GAabbreviate* [34,43]. The central cost reduction function was suggested by Yarkoni [40] for multidimensional measures. The adapted version for unidimensional instruments is:

$$Cost=Ik+1-R^{2}$$ (8)

where *I* represents a fixed item cost and *k* represents the number of items retained by the GA in any given iteration. *R*^2^ is the amount of variance accounted for by a linear combination of individual item scores. By varying *I*, a test developer can “place greater or less emphasis on the brevity of the measure relative to its comprehensiveness” [40](p182). High values of *I* result in a relatively brief instrument, since the cost of each additional item exceeds the costs caused by a loss in explained variance. Low values of *I* lead to a comparatively longer version because maximizing the amount of explained variance is more strongly weighted than shortening the measure. The basic idea behind GA is to lower the “redundancy within a scale, and therefore reducing the items to the substrate that does best in capturing the traits of interest” [28](p195). In the present case, we manipulated *I* (keeping all other arguments default) in order to get short scales with 25, 20, and 15 items.

## Results

**Fig 2:**
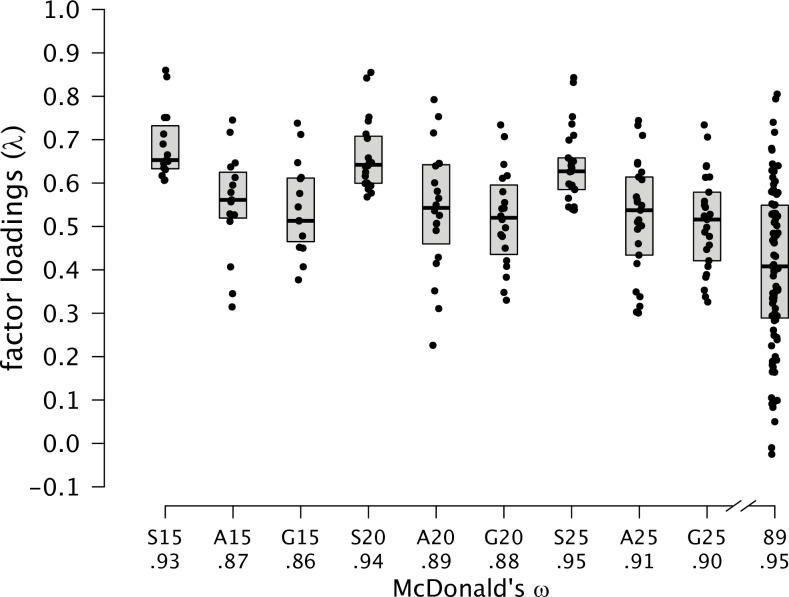
Distribution of Factor Loadings for the Original and Shortened Versions. A boxplot covers the interquartile range; the solid line refers to the median. Letters indicate the item selection algorithm: S = *Stepwise Confirmatory Factor Analysis*; A = *Ant Colony Optimization*; G = *Genetic Algorithm;* Numbers refer to the number of items in the abridged version; 89 = Original version with 89 items.

**Fig 3:**
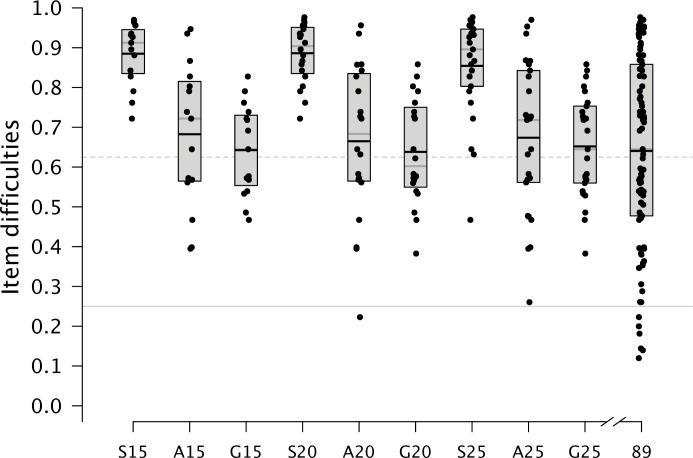
Distributions of the Item Difficulties of the Original and Shortened Versions. A boxplot covers the interquartile range; the solid line within a boxplot refers to the median. The gray solid line points out the guessing probability; the gray dashed line refers to the optimum item difficulty of .625. Letters indicate the item selection algorithm: S = *Stepwise Confirmatory Factor Analysis*; A = *Ant Colony Optimization*; G = *Genetic Algorithm;* Numbers refer to the number of items in the abridged version; 89 = Original version with 89 items. The horizontal gray line indicates the guessing probability (*P*(*x*) = .25).

**Fig 4:**
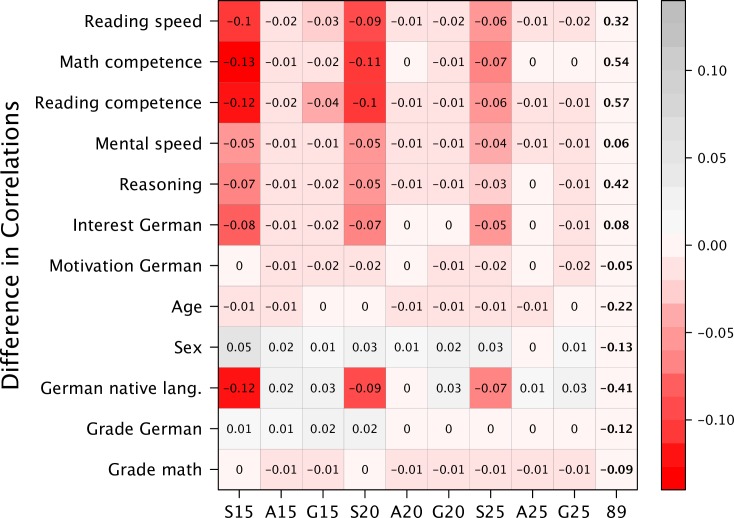
Correlation of the Original and Shortened Versions to Covariates. Letters indicate the item selection algorithm: S = *Stepwise Confirmatory Factor Analysis*; A = *Ant Colony Optimization*; G = *Genetic Algorithm;* Numbers refer to the number of items in the abridged version; 89 = Original version with 89 items.

Table 2 and Figs 2–4 summarize the main results concerning: a) model fit to detect deviations from unidimensionality, b) reliability estimates, c) discriminatory power at different points of the ability distribution, and d) changes in relations with the covariates. The selection strategies (i.e., SCOFA, ACO and GA) were evaluated in comparison to the original 89 item version on these four criteria. The first criterion dealt with the internal structure of the measures. For the PPVT-NEPS with 89 items a unidimensional model provided satisfactory fit (χ^2^_WLSMV_ = 22,944.4, *df* = 3,827, CFI = .933, RMSEA = .019), even though the CFI was slightly below the suggested cutoff-value of .95 [54]. Independent of the item selection strategy, all abbreviated versions had good absolute (RMSEA < .025) and incremental fit indices (CFI > .97; see Table 2). The abbreviated versions slightly outperformed the long version in terms of CFI. Thus, shortening did affect the unidimensional structure of the measure weakly and positively.

**Table 2:** Model Fit of Original and Shortened Versions.

Model	χ^2^_WLSMV_	*df*	CFI	RMSEA
SCOFA|15	410.94	90	.993	.016
ACO|15	606.62	90	.986	.020
GA|15	1,006.44	90	.979	.027
SCOFA|20	777.51	170	.990	.016
ACO|20	1,410.10	170	.979	.022
GA|20	1,576.96	170	.977	.024
SCOFA|25	1,409.38	275	.986	.017
ACO|25	1,923.25	275	.978	.020
GA|25	2,482.45	275	.973	.024
89	22,944.35	3,827	.933	.019

With respect to the models: Letters indicate the item selection algorithm: SCOFA = *Stepwise Confirmatory Factor Analysis*; ACO = *Ant Colony Optimization*; GA = *Genetic Algorithm;* Numbers refer to the number of items in the abridged version; 89 = Original version with 89 items. WLSMV = *Weighted Least Squares Mean and Variance adjusted*. CFI = Comparative Fit Index. RMSEA = Root Mean Square Error of Approximation.

Sufficiently high factor loadings have also been found as a necessary prerequisite for the correct interpretation of cutoff values for model fit [58]. Hence, the second criterion we considered was the distribution of factor loadings. Fig 2 gives the distribution of the factor loadings as well as McDonald’s *ω* (see formula 4) as a reliability estimate in a tau-congeneric measurement model. For both ACO and GA versions, the average factor loading was above .50 and, thus, noticeably higher than the mean loading in the original version (*M* = .41). Because reliability is overestimated in large item sets, the distribution and the average factor loadings draw a more comprehensive picture of the reliability. In comparison to the factor loadings of the original version that ranged between–.03 and .81, all abridged versions provided a higher average factor loading. Because SCOFA removed items with the lowest factor loadings, this selection algorithm, as expected, outperformed both ACO and GA in terms of model fit. Nevertheless, even for the extremely shortened 15-item versions, McDonald's *ω* was still very high for ACO (.90) and GA (.86). Furthermore, there were no systematic differences between ACO and GA in terms of factor saturation.

In order to assess the extent to which shortening affects the discriminative power of the measure, the distribution of item difficulties was plotted for the initial and the shortened versions (Fig 3). The item difficulties of PPVT-NEPS covered a broad range from .12 to .98 with an average close to the point of maximal information (*M* = .64). SCOFA selected items with a high proportion of correct responses resulting in an easy measure that would be suited to discriminate in the lower part of the ability distribution, but not across the whole ability distribution. Both ACO and GA versions covered a broader range of item difficulties with an average close to .625 (see solid black lines within the boxplots in Fig 3); extremely easy or extremely hard items tended to be removed. Again, there are no apparent differences between ACO and GA.

The last criterion refers to the aspect of validity expressed as manifest correlations between the sum score and constructs of the same or different scope. The last column of Fig 4 reports the zero-order correlations between the long version and selected covariates. The remaining columns give the differences in correlations between the abbreviated and PPVT-NEPS with covariates, where deeper shadings of red represent a loss in validity of the shortened version (see online supplement for a colored version). The largest deviations existed for the SCOFA versions, especially the shorter versions had substantial differences in correlations (15 items: *max*(Δ*r*) *=* .13; 20 items: *max*(Δ*r*) *=* .11). For ACO and GA the differences were much smaller with a maximum difference of .04 (GA 15-items). Overall, for both metaheuristics the differences in correlations are small and range around 0. Hence, the validity of the shortened measures in terms of correlations with covariates was not compromised. Comparing ACO with GA in regards to the four criteria, there was no systematic difference detectable. Bearing in mind that ACO finds a suitable, but not necessarily the best solution, the variation in the quality of solutions seems to be larger within ACO than between ACO and GA.

## Discussion

With the reign of large-scale, longitudinal, and multivariate assessment approaches in psychological, educational, and sociological research, the demand for psychometrically sound short scales has increased [59]. Reviewing the current practice, the methods that are often used to abbreviate existing measures—such as selecting the items with the highest factor loadings—are easy implemented, but poorly guided heuristics. Finitely terminating algorithms and iterative methods are often either overly simplistic or computationally demanding, depending on their functional complexity. In recent years, automated algorithms that are inspired by natural mechanisms, such as evolution or the foraging behavior of “eusocial” animals such as ants, have been proposed [33]. In the current study, we selected two metaheuristics (i.e., ACO and GA) out of a set of effective heuristics that have been developed recently in computational sciences. Among these alternative methods are *particle swarm optimization*, which try to systematically move particles around in the search-space to move the swarm toward a better solution. Similarly, *Tabu search* procedures are local search strategies that examine the neighborhood of a current solution while avoiding solutions that have already been explored [60]. *Artificial bee colony optimization* is another metaheuristic that mirrors the hierarchically organized behavior of different functional groups in a bee hive (i.e., employed bees, onlookers, and scouts). None of these approaches is superior *per se*; rather the effectiveness depends on the optimization problem at hand. In the same way bees and ants are optimally adapted to their environment and to their respective tasks, different heuristics are favorable for different optimization problems. With the current study, we added to the emerging literature in this field [30,32,40] showing the superiority of such metaheuristics in comparison to simple selection strategies. We demonstrated that metaheuristics are powerful tools that select item sets fulfilling several conditions (model fit, factor saturation, etc.). In contrast, stepwise reduction of the item pool with a single criterion comes at a cost. The implemented stepwise algorithm that maximizes factor loadings produced highly reliable short versions that were not valid (i.e., substantially lower correlations to covariates in comparison to the long form). The reason for this has passed into psychometric theory as *attenuation paradox* [61]. The paradox describes the fact that increasing item intercorrelations (i.e., internal consistency or reliability) of a test beyond a certain threshold will not increase its construct validity, but on the contrary will have adverse effects. This notion seems to contradict classical principles of test construction, but the underlying mechanism becomes more transparent if one looks at it from an informational perspective. If items are highly correlated, they are also highly redundant, which means that the gain of information by adding further homogenous items to a test is low. This redundancy is also reflected in the correlation matrices; for example, the tetrachoric correlation matrix of the 15-item version selected with SCOFA had an average of .51 (range: .33–.71), in comparison to the 15-item GA solution: *M* = .34 (range: .11–.56). In the present case, the high factor loadings are combined with low item difficulties which exacerbates the shortcomings—a problem also discussed by Loevinger [61,62]. For instance, SCOFA discarded difficult items from the beginning, resulting in a mean item difficulty of .92 (range: .83–.98), whereas GA provided a much larger range of item difficulties (.47–.83) around an average item difficulty (*M* = .64) near a theoretical point of maximal discrimination. From an informational perspective, items that are solved by nearly all participants have little to offer. In fact, individual differences in such items might shift from the accuracy of responses to response times, imposing the additional threat of a shifting the validity of the measure. In the present context, instead of reflecting the initial measurement intention of measuring receptive vocabulary, the SCOFA short measure might be more indicative of mental speed. Taken together, a test conveys more information—and can thus be a more valid measure of a construct—if more heterogeneous and moderately difficult items are compiled. One important conclusion from these results is that an item selection procedure might go astray with respect to criteria that are not explicitly considered. In the present case, the SCOFA procedure excels in what it explicitly focuses on, but fails with respect to many other criteria.

Both metaheuristic approaches do much better so we next turn to a comparison of these approaches. Please remember that ACO used a sophisticated optimization function and was computationally very demanding, whereas the GA used an unspecific cost-reduction function with a tradeoff between number of items and the amount of explained variance and was computationally simple. Put differently, we compare a theoretically derived optimization function that tackles important issues in short scale construction with an out-of-the-box optimization function. The implicit question that arises is to what extent item selection requires expertise from test developers versus what can be accomplished by machines. In the present case, the somewhat discomforting answer (for all human test developers) is that both ACO and GA algorithm compiled short versions that had, in comparison to the long version, the same factor structure, were reliable, sensitive in the intended ability band, and also valid in terms of maintenance of correlations with other variables. We expected that the tailored optimization function of ACO would compile abridged versions that outperform the ones constructed with a “dumb” and quick optimization algorithm. Surprisingly, this was not the case. The GA algorithm was much more time efficient (10 min. vs. > 24 hours for ACO).

Two intriguing questions arise with this finding. First, under what circumstances do ACO and GA produce different results, and second, in which instances is it advisable to use a metaheuristic with an unspecific default optimization function. To answer the first question, it is important to understand how the algorithms work. GA reduces the item set while explaining a maximum of individual differences in the long version, which results in maximizing the correlation between the abbreviated and the original scale. If the correlation is close to unity, the risk of diverging correlations is minimal within a nomological network or deviations in the shape of the score distributions. The ACO algorithm tries to find a compromise between several optimization criteria: a) model fit, b) factor saturation, c) sensitivity, and d) validity. The specified criteria also tend to develop short versions that are highly correlated to the long version. As a consequence, both GA and ACO develop along the same line, which is why they show very similar results. It is likely that the metaheuristics produce different results, when initial model fit is poor due to unspecified residual correlations between the items. Similarly, a more complex factor structure of the model that is not properly reflected in the measure (as often encountered in self-report measures) might cause stronger contrasts between metaheuristics pursuing different goals. In such instances, ACO could identify item samples that adhere to psychometric principles (i.e., reliability, validity), whereas GA fails due to its adherence to a flawed initial model.

This leads to the second question: when it is sensible to use an unspecific optimization algorithm? One could argue that GA presupposes that “the total score of the long scale is a valid representation of the underlying construct” [32](p65). This assumption is true in the present case of a unidimensional measure of maximal behavior (see also the model fit and distribution of factor loadings in the long version). However, this prerequisite is often not met. For example, in personality assessment, which mainly relies on measures of typical behavior, strict psychometric testing procedures, such as CFA modeling, usually fail for broad measures. The downside of the fact that GA creates short versions that closely match the long version is that it also reproduces its potential shortcomings. For example, the default GA optimization procedure was tied to the long scale, and hence, is not able to maximize the prediction of relevant outcomes. Therefore, as a recommendation for practitioners, we would advise against the use of GA (without an optimization function) in early stages of test development. We would generally advise against use of GA without optimization functions for measures with suboptimal model fit.

The results of this study also point to a more general issue in test construction that is heavily neglected in the scientific discourse: the process of *item sampling* and its influence on assessment. Whereas *person sampling* is an important topic in the context of generalizability, the influence of sampling indicators of a target construct on the results of scientific inquiry is often not perceived to be an issue. In the construction and validation of psychological measures, it is common practice to assume that items are drawn from a theoretically infinite item universe [13,57,63]. However, this assumption is not descriptive of reality. Loevinger [64](p147) and other psychometricians in the 1960s [65] pointed out that the random sampling assumption of items is unrealistic because test development is “almost invariably expert selection rather than sampling”. Accordingly, the results of the present study demonstrate the large extent to which reliability and validity are affected by the compilation of item sets. Please note that issues of item sampling are not restricted to compiling short-forms, but also apply to deriving the initial long versions. In many large-scale educational studies, such as PISA (*Programme for International Student Assessment*), several hundred items are administered in complex multiple matrix sampling designs [66], thus ensuring sufficient content coverage of the constructs. Obviously, building, implementing, and maintaining a large item database is expensive and time-consuming. Furthermore, multiple matrix designs require very large person samples. Because these conditions are rarely met in empirical educational studies, it is even more important to take item-sampling issues into account [67].

In their overview article “on the sins of short-form development”, Smith et al. [11] correctly pointed out that short versions should be tested empirically with an independent sample in order to show that the short form has adequate overlapping variance with the full form (sin #4), reproduces the factor structure (sin #5), has validity (sin #7), *et cetera*. Thus, the correct and adequate test would be to administer the newly compiled short version and to compare the results to the original version. However, if one thinks about the mechanisms that could change the short measure’s parameters in real testing, either these are methodological artifacts, such as item position effects, or undesirable test taking effects, such as fatigue or loss in motivation. These issues, if they apply at all, tend to affect the original version with many items stronger than in a shortened measure. Therefore, we think that in a representative sample, it is likely that the results are similar or unbiased. Another limitation is that we restricted our examination to only a few important aspects of test construction (e.g., model fit, reliability); other aspects such as measurement invariance across context variables such as sex or ethnicity were not included. Please keep in mind that the GA was also “blind” with respect to the four criteria we considered in this study and, nevertheless, produced efficient and psychometrically sound solutions. This shortcut strategy seems to work as long as the original version already features the intended quality. If this is not case, it would be necessary to incorporate additional criteria into the optimization function of metaheuristics so that the short version—given a sufficient large item pool—becomes an even better measure than the original one.
